# IDENTIFYING CARE TRANSITIONS NEEDS EXPERIENCED BY OLDER ADULTS AND CAREGIVERS HOSPITAL TO HOME

**DOI:** 10.1093/geroni/igad104.3404

**Published:** 2023-12-21

**Authors:** Claire Regan, Karen Hirschman, Molly McHugh, Onome Osokpo, Kathleen Mccauley, Elizabeth Shaid, Mary Naylor

**Affiliations:** University of Pennsylvania, Philadelphia, Pennsylvania, United States; University of Pennsylvania, Philadelphia, Pennsylvania, United States; University of Pennsylvania, Philadelphia, Pennsylvania, United States; University of Pennsylvania, Philadelphia, Pennsylvania, United States; University of Pennsylvania, Philadelphia, Pennsylvania, United States; University of Pennsylvania, Philadelphia, Pennsylvania, United States; University of Pennsylvania, Philadelphia, Pennsylvania, United States

## Abstract

Transitions from hospital to home are a fragile period for older adults, associated with increased risks for hospital readmission and mortality. The Transitional Care Model (TCM), a multi-component, advanced practice registered nurse (APRN) led, team-based care intervention designed to support older adults and their family caregivers during this transition, has consistently demonstrated improvements in both patients’ health outcomes while lowering care costs. This secondary analysis of data generated during a large-scale replication trial of the TCM conducted in three U.S. healthcare systems (ClinicalTrials.gov: NCT04494295) was designed to identify the key challenges experienced by APRNs in implementing TCM’s intervention protocol as designed. Data from multiple sources (i.e., case studies, case presentations, APRN documentation related to 184 intervention group patients) collected from September 2020 through August 2022 were analyzed using qualitative content analysis. Across sites, the top challenges in implementing the TCM and percentage reporting these issues were: social determinants of health (SDoH, 23%); complexity of care (15%); patient engagement (14%); and communication with patients/families (12%) and with providers (10%). Non-Veteran Affairs (VA) hospitals faced a higher number of SDoH challenges such as delays in accessing care and supporting non-English speaking patients, while VA hospitals had a higher number of patients with housing safety concerns. The VA sites also had a slightly higher percent of complexity of care (15%) challenges compared to non-VA sites (12%). Findings offer insight into enhancing the design of transitional care interventions by targeted challenges associated with SDoH, complexity of care, engagement, and communication.

